# Therapeutic strategies for idiopathic granulomatous mastitis: an umbrella review of systematic reviews and meta-analyses

**DOI:** 10.3389/fmed.2026.1852206

**Published:** 2026-06-03

**Authors:** Shuyu Yao, Leilai Xu

**Affiliations:** Department of Breast Surgery, The First Affiliated Hospital of Zhejiang Chinese Medical University (Zhejiang Provincial Hospital of Chinese Medicine), Hangzhou, China

**Keywords:** corticosteroids, idiopathic granulomatous mastitis, methotrexate, recurrence, surgery, systematic review, umbrella review

## Abstract

**Background:**

Idiopathic granulomatous mastitis (IGM) is a rare chronic inflammatory breast disease characterized by a prolonged clinical course with recurrent episodes, and the optimal treatment remains controversial. Multiple systematic reviews and meta-analyses have evaluated medical, surgical, and combined treatment strategies, but their conclusions remain inconsistent.

**Methods:**

We systematically searched PubMed, Embase, the Cochrane Library, and Web of Science for systematic reviews or meta-analyses evaluating remission, recurrence, and safety in patients with IGM or related granulomatous mastitis. The search period extended from database inception to 13 March 2026. The methodological quality of the included reviews was assessed using AMSTAR-2. Additionally, the overlap of primary studies across the reviews was evaluated using a citation matrix and the Corrected Covered Area (CCA).

**Results:**

Six reviews published between 2017 and 2026 were included, comprising five conventional systematic reviews/meta-analyses and one network meta-analysis. Overall methodological quality was suboptimal, with three reviews rated as low and three as critically low. Across the included reviews, steroid-based combination therapy showed the most consistent favorable signal, particularly for recurrence reduction. In the network meta-analysis, triple therapy consisting of surgery, local steroid injection, and systemic corticosteroids ranked most favorably for recurrence control. Local steroid therapy and methotrexate-based therapy also appeared promising, particularly as better-tolerated alternatives or adjuncts to systemic corticosteroids. In contrast, systemic corticosteroid monotherapy and conservative approaches appeared less favorable for long-term recurrence control. Across the six reviews, 97 unique primary studies and 113 total study occurrences were identified, yielding a CCA of 3.30%, indicating slight overlap.

**Conclusion:**

Steroid-based combination therapy showed the most consistent favorable signal, particularly for recurrence reduction. Local steroid therapy and methotrexate-based therapy also appeared promising in selected clinical contexts. However, the overall certainty of the evidence remains limited because of methodological weaknesses in the included reviews and the predominantly observational nature of the underlying literature. Further high-quality comparative research is needed before firm treatment recommendations can be established.

**Systematic review registration:**

PROSPERO, identifier [CRD420261337622].

## 1 Introduction

Idiopathic granulomatous mastitis (IGM) is a rare, benign, chronic inflammatory breast disease characterized by granulomatous inflammation centered on the lobules. Although non-malignant, it often follows a prolonged and recurrent clinical course, with manifestations such as breast mass, pain, abscess formation, fistulae, and inflammatory changes that may mimic infection or malignancy (1). Because of its uncertain etiology, variable presentation, and tendency to recur, IGM remains a challenging condition in clinical practice (2).

The optimal treatment of IGM remains controversial (3, 4). Reported management strategies include observation, drainage, antibiotics, systemic corticosteroids, local steroid therapy, surgery, methotrexate, and various combined regimens (2). In recent years, multiple systematic reviews and meta-analyses have attempted to synthesize evidence on these approaches. However, their conclusions have not been fully consistent, partly because of differences in study populations, intervention classifications, outcome definitions, and methodological rigor (3, 5).

Several issues complicate the interpretation of the existing review-level evidence. Some reviews are restricted to histopathologically confirmed IGM, whereas others include broader granulomatous mastitis (GM) or granulomatous lobular mastitis (GLM) populations (2). In the present review, IGM refers to histopathologically confirmed granulomatous inflammation centered on the breast lobules after exclusion of specific infectious, autoimmune, malignant, or other identifiable causes. GM is used as a broader descriptive term that may include idiopathic as well as secondary granulomatous inflammatory breast diseases. GLM is commonly used in the literature to describe lobule-centered granulomatous inflammation and substantially overlaps with IGM, although terminology varies across studies. Recurrent or refractory granulomatous mastitis refers to disease that recurs after initial treatment or shows an inadequate response to conventional medical or surgical management. In addition, remission, recurrence, and safety outcomes are not uniformly defined, and treatment modalities vary from single medical therapies to multimodal strategies and network-based treatment nodes (4). Moreover, the methodological quality of the available reviews and the degree of overlap of primary studies across reviews have not been comprehensively evaluated in a unified framework. As a result, clinicians may find it difficult to determine which treatment strategies are supported by the most consistent and clinically meaningful evidence (2, 6).

An umbrella review is well-suited to address these gaps because it enables the synthesis and critical appraisal of published systematic reviews and meta-analyses at the review level (7). Such an approach is particularly relevant for IGM, where multiple overlapping reviews now exist, but the overall strength, consistency, and interpretability of the evidence remain uncertain (8).

Therefore, the present umbrella review aimed to synthesize and critically appraise published systematic reviews and meta-analyses on treatment strategies for idiopathic granulomatous mastitis. Specifically, we summarized evidence on remission, recurrence, and safety; evaluated methodological quality using AMSTAR-2; and identified the treatment strategies showing the most consistent favorable signal while highlighting the limitations and gaps in the current evidence base.

## 2 Methods

### 2.1 Review protocol

This umbrella review aimed to synthesize and critically appraise the evidence on treatment strategies for idiopathic granulomatous mastitis (IGM). The review methodology was informed by methodological guidance for umbrella reviews (9) and followed the Preferred Reporting Items for Systematic Reviews and Meta-Analyses (PRISMA) 2020 statement (10). The protocol was prospectively registered with the International Prospective Register of Systematic Reviews (PROSPERO) (registration number: CRD420261337622).

### 2.2 Search strategy

To identify relevant systematic reviews and meta-analyses, comprehensive literature searches were conducted in PubMed, Embase, Web of Science, and the Cochrane Library. Search terms combined controlled vocabulary and free-text keywords related to the disease, treatment strategies, and review type, including “idiopathic granulomatous mastitis,” “granulomatous mastitis,” “granulomatous lobular mastitis,” “treatment,” “therapy,” “surgery,” “corticosteroids,” “local steroid,” “methotrexate,” “systematic review,” “meta-analysis,” and “network meta-analysis.” The detailed search strategy for each database is presented in Supplementary Table 1.

Searches were limited to English-language publications from database inception to 13 March 2026. Manual reference screening of included reviews was additionally performed to ensure completeness. Gray literature and non-peer-reviewed sources were excluded.

### 2.3 Eligibility criteria

Systematic reviews, meta-analyses, and network meta-analyses were included if they: (1) explicitly described systematic methods for literature identification, selection, and synthesis; (2) focused on patients with idiopathic granulomatous mastitis or granulomatous mastitis/granulomatous lobular mastitis in which therapeutic outcomes were the main focus; (3) evaluated one or more treatment strategies, including surgery, systemic corticosteroids, local steroid therapy, methotrexate, steroid-based combination therapy, drainage, antibiotics, observation, or other conservative or multimodal approaches; and (4) reported clinically relevant outcomes such as complete remission, clinical remission, response, recurrence, adverse events, treatment failure, or treatment discontinuation.

Reviews that exclusively examined non-therapeutic topics, diagnostic accuracy, imaging features, pathology, etiology, or other non-treatment-related aspects were excluded unless treatment-specific analyses were separately reported. Narrative reviews, expert opinions, letters, conference abstracts, case reports, and original primary studies were also excluded.

Primary outcomes of interest included remission-related outcomes (complete remission, clinical remission, treatment response), recurrence, and safety outcomes (adverse events, severe adverse events, treatment discontinuation, or complications). Where available, data on comparative treatment efficacy and pooled outcome estimates were also extracted.

### 2.4 Screening and selection process

All records retrieved were imported into reference management software for screening and deduplication. Two independent investigators (Leilai Xu and Shuyu Yao) screened titles and abstracts according to the inclusion criteria. Full texts of potentially eligible studies were then assessed independently by the same two reviewers (Leilai Xu and Shuyu Yao). Discrepancies were resolved through discussion and consensus to ensure consistency. The overall study selection process was documented using a PRISMA flow diagram. For records with potentially eligible titles and abstracts but unavailable full texts, additional manual searching and institutional access attempts were undertaken; however, some reports remained non-retrievable because no accessible full-text version could be obtained.

### 2.5 Data extraction

A standardized extraction template was used to capture review characteristics, including first author, publication year, journal, databases searched, search end date, review type, number of included primary studies, total sample size, target population, intervention categories, outcomes, key findings, and quality appraisal methods. Each included review was independently extracted by two reviewers (Leilai Xu and Shuyu Yao) to ensure accuracy.

In addition, quantitative and qualitative findings were extracted whenever available, including pooled effect estimates, pooled proportions, 95% confidence intervals, heterogeneity measures, and authors’ main interpretations regarding remission, recurrence, and safety. Special attention was paid to whether reviews were restricted to idiopathic granulomatous mastitis or included broader granulomatous mastitis populations. Disease labels and definitions, including IGM, GM, GLM, and recurrent or refractory disease, were also extracted and considered when interpreting the applicability of findings across reviews.

### 2.6 Quality appraisal and certainty of evidence

Methodological quality of the included systematic reviews was evaluated using AMSTAR-2 (A MeaSurement Tool to Assess Systematic Reviews 2) (11), with particular attention to key domains such as protocol registration, comprehensiveness of the literature search, justification for study exclusion, risk-of-bias assessment, appropriateness of synthesis methods, and consideration of bias when interpreting findings. Each review was independently appraised by two reviewers, with disagreements resolved by discussion and consensus.

Because the present study was an umbrella review of published systematic reviews and meta-analyses rather than a *de novo* synthesis of primary studies, a formal *de novo* GRADE assessment for each clinical endpoint was not undertaken. Instead, the certainty and interpretability of the evidence were judged in light of the methodological quality of the included reviews, the consistency of findings across reviews, the degree of overlap among primary studies, and the limitations of the underlying evidence base.

### 2.7 Data synthesis

Given the expected heterogeneity in population definitions, intervention classifications, outcome measures, and synthesis methods across the included reviews, a narrative synthesis approach was used (12). Data were organized by treatment category, including systemic corticosteroids, local steroid therapy, surgery, steroid-based combination therapy, methotrexate-based therapy, and other conservative or multimodal strategies. Quantitative findings reported in the included meta-analyses, including pooled estimates of remission, recurrence, and adverse events, were extracted and summarized descriptively to illustrate the direction and magnitude of treatment effects.

To determine the degree of overlap among the included reviews, a citation matrix of the primary studies was constructed, and the corrected covered area (CCA) index (13) was calculated. Overlap was explicitly considered in the interpretation of the independence of the evidence base and in our decision not to undertake *de novo* re-pooling of quantitative estimates across reviews.

### 2.8 Ethical approval

Ethical approval was not required, as this study was a review of published studies and no human participants were directly involved.

## 3 Results

### 3.1 Search results

**FIGURE 1:**
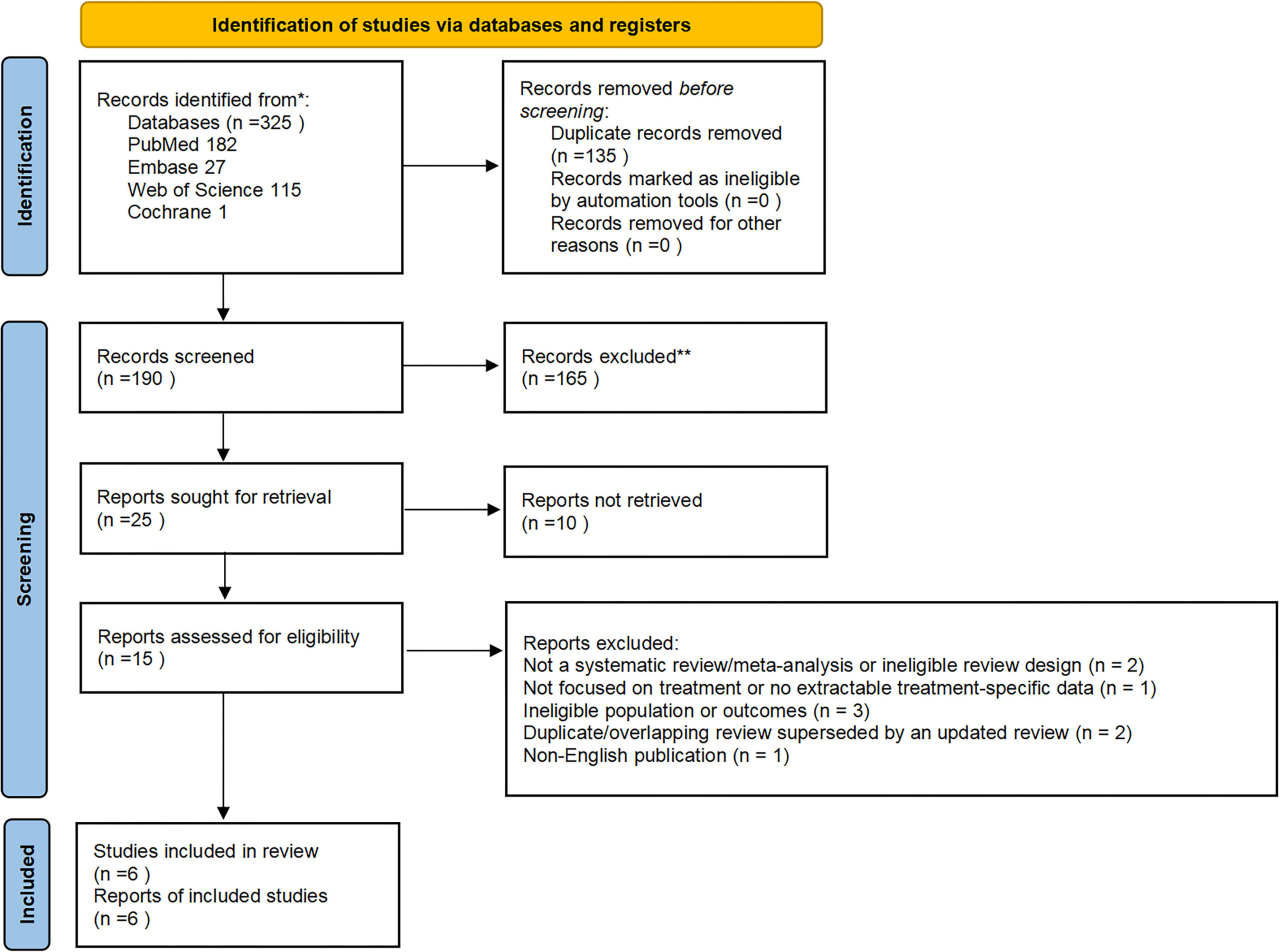
PRISMA 2020 flow diagram of the study selection process.

A total of 325 records were identified through database searching, including 182 from PubMed, 27 from Embase, 115 from Web of Science, and 1 from the Cochrane Library. After removal of 135 duplicate records, 190 records remained for title and abstract screening. Following initial screening, 25 reports were sought for retrieval, of which 10 were not retrieved. These non-retrieved reports mainly consisted of records for which no accessible full-text version could be obtained despite additional manual searching and institutional access attempts. The remaining 15 full-text articles were assessed for eligibility, and nine were excluded for predefined reasons, including ineligible review design, lack of treatment-specific data, ineligible population or outcomes, overlapping reviews superseded by updated versions, and non-English publication. Ultimately, six systematic reviews and meta-analyses (5, 6, 14–17) were included in this umbrella review. The study selection process is presented in Figure 1.

### 3.2 Characteristics of included reviews

The six included reviews were published between 2017 and 2026 and consisted of five conventional systematic reviews and meta-analyses and one network meta-analysis. The number of primary studies included in each review ranged from 8 to 65, and the reported sample sizes ranged from 559 to 3,222 participants.

The included reviews exhibited substantial heterogeneity. Three reviews focused on histopathologically confirmed idiopathic granulomatous mastitis (IGM), whereas others included broader populations such as granulomatous mastitis (GM) or granulomatous lobular mastitis (GLM). The interventions assessed across the reviews included systemic corticosteroids, local steroid therapy, surgery, steroid-based combination therapy, methotrexate-based therapy, and conservative or multimodal approaches. The main outcomes reported were remission- or response-related outcomes, recurrence, and treatment-related adverse events.

In terms of review focus, Lei et al. (14), Sarmadian et al. (15) provided broad overviews of multiple treatment strategies, whereas Godazandeh et al. (6) focused mainly on corticosteroid- and surgery-based approaches. Zhang et al. (16) specifically evaluated local steroid administration, Han et al. (5) focused on methotrexate-based therapy, and Zhou and Xu (17) performed a network meta-analysis comparing multiple treatment nodes. Detailed characteristics of the included reviews are summarized in Table 1.

**TABLE 1:** Characteristics and methodological quality of included reviews.

References	Review type	Included studies (participants)	Population	Interventions/ comparators	Main outcomes	Quality appraisal in original review	Registra- tion reported	AMSTAR-2 overall confidence*	Main findings
Lei et al. (14)	Systematic review and meta-analysis	15 studies (704)	Patients with IGM after exclusion of other causes of granulomatous mastitis	Surgery, oral steroids, topical steroids, observation, and combination regimens including MTX or prolactin-lowering agents	Complete remission/resolution, recurrence	NR	NR	Critically Low	Surgery, with or without steroids, was associated with higher complete remission and better recurrence control than steroid monotherapy; however, the evidence was based on heterogeneous non-randomized studies
Godazandeh et al. (6)	Systematic review and meta-analysis	12 studies (559)	Histopathologically confirmed IGM	Steroid-only, surgery-only, steroid plus surgery	Recurrence, side effects	NOS	NR	Critically low	Steroid plus surgery was associated with lower recurrence and fewer side effects than steroid monotherapy; however, the available evidence was limited and largely observational
Zhang et al. (16)	Systematic review and meta-analysis	Eight studies (613)	Histopathologically confirmed IGM	Local steroid administration versus systemic therapy or surgery	Response rate, recurrence, side effects, pain	Cochrane Collaboration risk-of-bias tool	PROSPERO reported	Low	Local steroid administration was associated with a higher response rate and fewer side effects than comparator treatments; however, no significant difference in recurrence was observed
Sarmadian et al. (15)	Systematic review and meta-analysis	65 studies (3,222)	Patients with granulomatous mastitis (GM)	Systemic steroids, topical steroids, antibiotics, MTX, observation, drainage, excision, and combination therapies	Recurrence	Quality assessment checklist for prevalence studies (18)	NR	Critically low	Combination therapy was associated with lower recurrence than several single-modality approaches; however, recurrence estimates varied substantially across treatment types and study populations
Zhou and Xu (17)	Systematic review and network meta-analysis	19 studies (1,095)	Patients with GLM/IGM	Observation, systemic steroids, local steroid injection, surgery, and multiple combined regimens	Recurrence	NOS	PROSPERO reported	Low	Triple therapy consisting of surgery, local steroid injection, and systemic steroids was associated with the most favorable recurrence ranking; however, the findings were derived from indirect comparisons in a network meta-analysis
Han et al. (5)	Systematic review and meta-analysis	Nine studies (735)	Histopathologically confirmed IGM	MTX monotherapy and MTX-based combination therapy	Complete remission, recurrence, severe side effects leading to MTX discontinuation	NOS	PROSPERO reported	Low	MTX-based therapy was associated with favorable remission and tolerability outcomes; however, the evidence was heterogeneous and largely derived from observational studies

IGM, idiopathic granulomatous mastitis; GM, granulomatous mastitis; GLM, granulomatous lobular mastitis; NOS, Newcastle–Ottawa Scale; NR, not reported.

*AMSTAR-2 overall confidence was assigned by the authors of the present umbrella review.

### 3.3 Methodological quality of included reviews

Methodological quality was assessed using AMSTAR-2. Overall, the methodological quality of the included reviews was suboptimal. Of the six reviews, three were rated low quality, and three were rated critically low quality.

The reviews by Han et al. (5), Zhou and Xu (17), Zhang et al. (16) were judged to have low overall confidence, whereas Lei et al. (14), Godazandeh et al. (6), Sarmadian et al. (15) were rated as critically low. The most frequent critical weakness across reviews was the absence of a list of excluded full-text studies, along with justification for each exclusion. Additional commonly observed limitations included a lack of protocol registration in some reviews, an incomplete assessment of the influence of risk of bias on pooled findings, and limited consideration of methodological weaknesses of primary studies when interpreting the results.

Despite these shortcomings, several of the more recent reviews reported comprehensive search strategies, duplicate screening and data extraction, formal risk-of-bias assessment tools, and appropriate statistical synthesis methods. The detailed AMSTAR-2 appraisal is provided in Supplementary Table 2.

### 3.4 Overlap of primary studies

A citation matrix was constructed to assess the overlap of primary studies across the included reviews. Across the six reviews, there were 97 unique primary studies and 113 total study occurrences. The corrected covered area (CCA) was 3.30%, indicating a slight degree of overlap among the included reviews.

This finding suggests that although several reviews addressed similar treatment questions, the overlap in the primary evidence was limited. Therefore, the conclusions of the included reviews cannot be considered entirely independent, but neither were they dominated by extensive redundancy of the same evidence base. The citation matrix is provided in Supplementary material.

Because even slight overlap may still introduce duplication of underlying evidence in umbrella reviews, the observed overlap was taken into account when interpreting consistency across reviews and when considering whether quantitative re-pooling would be methodologically appropriate.

### 3.5 Findings by treatment category

The main findings across treatment categories are summarized in Table 2.

**TABLE 2:** Summary of findings by treatment category across included reviews.

Treatment category	Relevant reviews	Remission findings	Recurrence findings	Safety findings	Overall interpretation	Major caveats
Systemic corticosteroids	Lei et al. (14); Godazandeh et al. (6); Sarmadian et al. (15); Zhou and Xu (17)	Systemic corticosteroids were associated with clinical remission in a substantial proportion of patients, although remission outcomes appeared less favorable than those reported for some combined strategies.	Recurrence after steroid monotherapy was generally higher than after steroid-based combination therapy.	Treatment-related adverse effects were more frequently reported with systemic corticosteroids than with local steroid approaches.	Systemic corticosteroids remain an important medical option, particularly for inflammatory control, but steroid monotherapy appears less effective for long-term recurrence prevention than selected combined regimens.	Definitions of remission varied across reviews; evidence was predominantly based on observational studies; some reviews included broader granulomatous mastitis or granulomatous lobular mastitis populations rather than strictly idiopathic granulomatous mastitis; adverse-event reporting was inconsistent.
Local steroid therapy	Zhang et al. (16); Sarmadian et al. (15); Zhou and Xu (17)	Local steroid therapy was associated with favorable response and remission-related outcomes in selected patients.	Recurrence was not consistently lower than all comparators in direct comparisons, although local steroid injection ranked favorably for recurrence control in the network meta-analysis.	Local steroid therapy was generally associated with fewer systemic adverse effects than systemic treatment; local complications were infrequently and inconsistently reported.	Local steroid therapy appears to be a promising and better-tolerated option, particularly for localized disease, although the evidence base remains limited.	The evidence base was relatively small; outcome definitions were inconsistent; one key review relied partly on indirect comparison and treatment ranking rather than direct head-to-head evidence; reporting of local adverse events was limited.
Surgery	Lei et al. (14); Godazandeh et al. (6); Sarmadian et al. (15); Zhou and Xu (17)	Surgery, with or without adjunctive therapy, was associated with high remission in several reviews, particularly in refractory or localized cases.	Recurrence after surgery alone was generally lower than with conservative approaches, but not necessarily lower than with optimized combined strategies.	Surgical morbidity, cosmetic burden, and patient-reported outcomes were not uniformly reported across reviews.	Surgery remains an effective option, especially for refractory or localized disease, but should not automatically be considered superior to all non-surgical strategies.	Surgical techniques and indications varied markedly; patient selection likely influenced outcomes; cosmetic and quality-of-life outcomes were rarely reported; comparative safety data were limited.
Steroid-based combination therapy	Lei et al. (14); Godazandeh et al. (6); Sarmadian et al. (15); Zhou and Xu (17)	Combination regimens involving corticosteroids and surgery were associated with favorable remission outcomes and were often among the best-performing strategies across reviews.	Combination therapy was consistently associated with lower recurrence than several single-modality approaches; triple therapy showed the best recurrence ranking in Zhou and Xu (17).	Safety appeared acceptable in the available reviews, although direct comparative safety data were limited.	Steroid-based combination therapy appears to provide the most consistent advantage across reviews, particularly for recurrence reduction.	Much of the evidence came from retrospective studies; combination regimens were heterogeneous across reviews; ranking in the network meta-analysis was based partly on indirect evidence; standardized safety reporting was lacking.
Methotrexate-based therapy	Han et al. (5); Lei et al. (14); Sarmadian et al. (15)	Methotrexate-based therapy was associated with favorable remission outcomes, particularly as an adjunctive or steroid-sparing strategy.	Recurrence appeared low in methotrexate-containing regimens in some reviews; Sarmadian et al. (15) reported particularly low recurrence with methotrexate plus corticosteroids.	Methotrexate was generally reported as well-tolerated, with relatively low rates of severe adverse events leading to discontinuation in Han et al. (5).	Methotrexate appears to be a useful adjunctive or steroid-sparing option, but the certainty of evidence remains limited.	Evidence was largely observational; methotrexate was used in heterogeneous clinical settings and combinations; direct comparative evidence was limited; long-term safety reporting was incomplete.
Conservative/ other strategies	Lei et al. (14); Sarmadian et al. (15); Zhou and Xu (17)	Observation and other conservative approaches showed variable remission outcomes and generally less favorable disease control than active intervention strategies.	Drainage and some conservative modalities were associated with relatively high recurrence in Sarmadian et al. (15); observation generally did not rank among the most favorable strategies.	Safety was poorly and inconsistently reported across reviews.	Conservative approaches may be appropriate in selected cases, but overall they appeared less favorable than active medical or combined treatment strategies for recurrence control.	These categories were highly heterogeneous and often poorly defined; patient selection criteria and follow-up duration were inconsistently reported; the absence of reported adverse events should not be interpreted as absence of treatment-related risk.

IGM, idiopathic granulomatous mastitis; GM, granulomatous mastitis; GLM, granulomatous lobular mastitis. “Relevant reviews” indicates which of the six included systematic reviews reported data for each treatment category. Remission, recurrence, and safety findings are summarized qualitatively based on the conclusions of the included reviews rather than re-pooled quantitative estimates. Specific pooled effect sizes, where available, should be interpreted in the context of each original review. Absence of reported adverse events does not imply absence of treatment-related risk.

#### 3.5.1 Systemic corticosteroids

Systemic corticosteroids were evaluated in Lei et al. (14), Godazandeh et al. (6), Sarmadian et al. (15), Zhou and Xu (17). Across these reviews, systemic corticosteroids were associated with clinical remission in a substantial proportion of patients, although remission outcomes appeared less favorable than those reported for some combined strategies.

Recurrence after steroid monotherapy was generally higher than after steroid-based combination therapy. In addition, treatment-related adverse effects were more frequently reported with systemic corticosteroids than with local steroid approaches. Overall, systemic corticosteroids remain an important medical option, particularly for inflammatory control, but steroid monotherapy appears less effective for long-term recurrence prevention than selected combined regimens.

#### 3.5.2 Local steroid therapy

Local steroid therapy was addressed primarily in Zhang et al. (16), and was also represented in Sarmadian et al. (15), Zhou and Xu (17). Across these reviews, local steroid administration was associated with favorable response and remission-related outcomes in selected patients. In direct comparisons, recurrence was not consistently lower than all alternative treatments, although local steroid injection ranked favorably for recurrence control in the network meta-analysis by Zhou and Xu (17).

Local steroid therapy was generally associated with fewer systemic adverse effects than systemic treatment, while local complications were infrequently and inconsistently reported. Taken together, these findings suggest that local steroid therapy is a promising and better-tolerated option, particularly for localized disease, although the evidence base remains limited.

#### 3.5.3 Surgery

Surgery was discussed in Lei et al. (14), Godazandeh et al. (6), Sarmadian et al. (15), Zhou and Xu (17). Surgery, either alone or in combination with adjunctive therapy, was associated with high remission rates in several reviews, especially in patients with refractory or localized disease.

Recurrence after surgery alone was generally lower than that reported for conservative approaches, but not necessarily lower than that observed with optimized combined strategies.

Safety outcomes related to surgery were not consistently reported. In particular, surgical morbidity, cosmetic burden, and patient-reported outcomes were poorly captured across reviews. Therefore, although surgery remains an effective option, it should not be assumed to be universally superior to all non-surgical approaches.

#### 3.5.4 Steroid-based combination therapy

Steroid-based combination therapy was reported in Lei et al. (14), Godazandeh et al. (6), Sarmadian et al. (15), Zhou and Xu (17). Combination strategies, particularly those involving corticosteroids and surgery, were associated with favorable remission outcomes and were repeatedly reported to reduce recurrence more effectively than several single-modality approaches.

Notably, in the network meta-analysis by Zhou and Xu (17), triple therapy consisting of surgery, local steroid injection, and systemic corticosteroids showed the most favorable recurrence ranking. Overall, steroid-based combination therapy appeared to offer the most consistent benefit, particularly for reducing recurrence, although the available evidence remained largely based on retrospective, heterogeneous studies.

#### 3.5.5 Methotrexate-based therapy

Methotrexate-based therapy was reported in Lei et al. (14), Sarmadian et al. (15), Han et al. (5). Across these reviews, methotrexate appeared to be associated with favorable remission outcomes, particularly when used as an adjunctive or steroid-sparing strategy. Recurrence was also low in some methotrexate-containing regimens, with Sarmadian et al. (15) reporting particularly low recurrence for methotrexate combined with corticosteroids.

In terms of safety, methotrexate was generally well-tolerated, and Han et al. (5) reported relatively low rates of severe adverse events leading to treatment discontinuation. Nevertheless, the underlying evidence remained largely observational, and methotrexate was used across heterogeneous clinical settings and in various combinations.

#### 3.5.6 Conservative and other strategies

Observation and other conservative strategies were described in Lei et al. (14), Sarmadian et al. (15), Zhou and Xu (17). These approaches showed variable remission outcomes and generally less favorable disease control than active intervention strategies. In Sarmadian et al. (15), drainage and several conservative modalities were associated with relatively high recurrence rates, whereas observation did not rank among the most favorable strategies in Zhou and Xu (17).

Safety reporting for conservative and miscellaneous strategies was limited and inconsistent. These categories were also highly heterogeneous and often poorly defined, making firm conclusions difficult. Overall, conservative approaches may be appropriate in selected cases, but they appeared less favorable than active medical or combined treatment strategies for recurrence control.

### 3.6 Overall synthesis of findings

Across the included reviews, steroid-based combination therapy showed the most consistent favorable signal, particularly for recurrence reduction. Local steroid therapy and methotrexate-based therapy also appeared promising, especially in terms of tolerability and their potential role as alternatives or adjuncts to systemic corticosteroids. In contrast, steroid monotherapy and conservative approaches appeared less favorable for long-term recurrence control.

However, these findings should be interpreted cautiously because the underlying evidence base was predominantly observational, the methodological quality of the included reviews was generally low or critically low, and important heterogeneity remained in population definitions, intervention classifications, and outcome reporting.

## 4 Discussion

In this umbrella review, we synthesized the available systematic reviews and meta-analyses on treatment strategies for idiopathic granulomatous mastitis (IGM) and related granulomatous mastitis populations. Three key findings emerged. First, steroid-based combination regimens demonstrated the most consistent favorable signal across the included reviews, particularly for recurrence reduction (15, 17). Second, local steroid therapy and methotrexate-based therapy also appeared promising, especially for tolerability and as alternatives or adjuncts to systemic corticosteroids (5, 15, 16). Third, systemic corticosteroid monotherapy and conservative approaches were generally inferior to selected combined regimens for long-term recurrence control (15, 17). Nevertheless, these conclusions must be interpreted with caution. The underlying evidence base is predominantly observational, the overall methodological quality of the included reviews is suboptimal, and substantial heterogeneity persists across patient populations, intervention classifications, and outcome definitions (5, 6, 14–17).

Systemic corticosteroids remain one of the most important medical treatment options for IGM, particularly for controlling active inflammation and relieving acute symptoms. In clinical practice, corticosteroids are often used as an initial medical strategy because they may reduce the inflammatory burden and avoid immediate surgical intervention in selected patients (19, 20). Nevertheless, the present umbrella review suggests that steroid monotherapy may be insufficient for durable long-term disease control, especially when recurrence is the primary endpoint. This pattern was observed across several included reviews, which generally reported less favorable recurrence outcomes with steroid monotherapy than with steroid-based combination regimens. In addition, systemic corticosteroids are associated with well-recognized adverse effects, which may limit treatment duration, patient adherence, and overall acceptability (21). Taken together, the current evidence supports the continued clinical relevance of systemic corticosteroids, but also highlights the limitations of relying on steroid monotherapy as a definitive long-term strategy.

One of the most consistent findings of this umbrella review was the apparent advantage of steroid-based combination therapy, especially combinations involving corticosteroids and surgery. Across multiple reviews, these combined strategies were associated with favorable remission outcomes and lower recurrence rates than several single-modality approaches (6, 14, 15, 17). A plausible explanation is that combined therapy may better address both the inflammatory and structural components of the disease. Corticosteroids may help suppress ongoing inflammatory activity, whereas surgery may help eliminate persistent localized lesions, sinus tracts, or tissue that has responded inadequately to medical treatment alone (21, 22). This interpretation is also supported by the network meta-analysis included in the present review, which found that triple therapy consisting of surgery, local steroid injection, and systemic corticosteroids had the most favorable recurrence ranking. Even so, this apparent advantage should not be overstated. The combination regimens evaluated across reviews were heterogeneous, patient selection was likely non-random, and much of the evidence came from retrospective studies (17). Therefore, although the overall signal favors steroid-based combination therapy, the magnitude and generalizability of this benefit remain uncertain.

Surgical treatment should also be interpreted beyond recurrence control alone. Because IGM often affects young women and may present with abscesses, fistulae, or deforming inflammatory lesions, surgical decision-making should balance disease clearance with postoperative breast appearance, patient satisfaction, quality of life, and long-term functional outcomes. These patient-centered outcomes were insufficiently captured in the included reviews, which limits the ability to compare surgical and non-surgical strategies from a patient-centered perspective. Recent primary evidence in refractory granulomatous mastitis has suggested that selected surgical techniques, such as rotational gland dissection, may achieve acceptable disease control while also reporting postoperative breast appearance, patient satisfaction, and long-term follow-up outcomes (23). Although this study was not eligible for inclusion in the present umbrella review because it was a single-center retrospective primary study, it supports the clinical need to incorporate cosmetic and patient-reported outcomes into future comparative studies of surgical treatment for IGM.

The findings regarding local steroid therapy are also clinically important. Compared with systemic corticosteroids, local steroid administration offers a more targeted approach, with the potential to reduce systemic exposure while maintaining therapeutic benefit in selected patients (21). Across the included reviews, local steroid therapy was associated with favorable response and remission-related outcomes, and was generally reported to cause fewer systemic adverse effects than systemic treatment (15). This is particularly relevant in IGM, where the disease often affects younger women and where long-term tolerability may strongly influence treatment choice (4). In the network meta-analysis, local steroid injection also ranked favorably for recurrence control (17). However, the current evidence is not yet sufficient to conclude that local steroid therapy is universally superior to systemic therapy. Direct comparative evidence remains limited, definitions of response and recurrence vary, and local adverse events were infrequently and inconsistently reported (4). For these reasons, local steroid therapy should be regarded as a promising strategy, especially for localized disease, but it still requires a more robust comparative evaluation. This issue may be particularly relevant in recurrent or refractory IGM, where treatment route and local disease control become especially important. Recent comparative evidence has suggested that intralesional steroid treatment may represent a useful option in selected recurrent or refractory cases (24), further supporting the need for more individualized treatment strategies in clinical practice.

Methotrexate-based therapy represents another noteworthy finding of this umbrella review. The included evidence suggests that methotrexate may serve as a useful adjunctive or steroid-sparing strategy, with favorable remission-related outcomes and generally acceptable tolerability (5). This is clinically meaningful because some patients experience recurrent disease, poor tolerance to corticosteroids, or an inadequate response to steroid monotherapy. In such settings, methotrexate may help reduce corticosteroid exposure while maintaining disease control (2). In the present review, methotrexate-containing regimens appeared to have low recurrence rates in some evidence syntheses, and one review reported relatively low rates of severe adverse events leading to discontinuation (5, 15). However, the evidence base remains limited and heterogeneous. Methotrexate was used across different clinical settings, at different doses, and in different combinations, and most supporting studies were observational rather than randomized (5, 15). Therefore, methotrexate should not be considered an established first-line standard, but rather a potentially valuable adjunctive option for carefully selected patients.

We intentionally summarized findings qualitatively rather than undertaking *de novo* re-pooling of quantitative estimates across the included reviews. Although the calculated overlap among reviews was slight (CCA 3.30%), some primary studies were represented in more than one review, and simple review-level re-pooling could therefore have introduced double-counting and misleadingly precise estimates. In addition, substantial heterogeneity remained in population definitions, treatment classifications, outcome measures, and follow-up duration across the included reviews. Under these circumstances, a *de novo* network meta-analysis was also not considered methodologically appropriate within the current umbrella review framework. The present study was designed to synthesize and critically appraise review-level evidence rather than reconstruct primary-study-level comparative estimates.

This review also highlights several important methodological and evidence-related limitations in the current literature. First, the methodological quality of the included reviews was generally low or critically low according to AMSTAR-2, with common weaknesses including lack of protocol registration, inadequate reporting of excluded studies, incomplete consideration of bias when interpreting results, and insufficient reporting of funding sources. These recurrent deficiencies reduce transparency and should be addressed more explicitly in future evidence syntheses in this field. Second, although the overlap analysis showed only a slight degree of overlap among primary studies, the underlying evidence base was still largely composed of small observational studies, which inherently limit causal inference. Third, substantial heterogeneity existed in disease definitions, clinical severity, and outcome reporting. Some reviews focused strictly on histopathologically confirmed IGM, whereas others included broader GM or GLM populations, and some primary studies further included recurrent or refractory cases. Because these terms were not consistently defined across the included reviews, differences in disease labels and clinical severity may have affected the comparability and applicability of the findings. Likewise, remission, response, and recurrence were inconsistently defined across reviews, and follow-up duration varied considerably. Fourth, treatment categories themselves were heterogeneous; even within the same nominal category, such as steroid-based combination therapy, the actual regimens differed across studies and reviews. Finally, patient-centered outcomes, including cosmetic burden, quality of life, functional outcomes, and patient preferences, were rarely reported. In addition, a formal *de novo* GRADE assessment was not undertaken because the present study synthesized review-level evidence rather than reconstructing endpoint-specific bodies of primary evidence. This should be taken into account when interpreting the certainty and clinical applicability of the conclusions.

Despite these limitations, the present umbrella review has several important strengths. This is among the few attempts to synthesize the secondary evidence on IGM treatment in a structured umbrella review framework. By integrating findings from systematic reviews, meta-analyses, and one network meta-analysis, this study provides a more comprehensive overview of the treatment evidence landscape than any single review alone. In addition, this review did not merely summarize treatment effects; it also assessed the methodological quality of the included reviews using AMSTAR-2 and quantified primary study overlap using a citation matrix and corrected covered area (CCA) analysis. These additional steps are important because they help distinguish between the apparent consistency of findings and the actual reliability of the underlying evidence base. The present review, therefore, contributes not only a clinical summary but also a critical appraisal of how much confidence can reasonably be placed in that summary.

From a clinical perspective, the current evidence suggests that steroid-based combination therapy may offer the most favorable balance for recurrence control. In contrast, local steroid therapy and methotrexate-based therapy may represent useful alternatives or adjuncts in selected settings. At the same time, the available evidence does not support overly strong recommendations regarding a universally superior strategy. Treatment decisions should still be individualized according to disease extent, symptom severity, lesion localization, prior treatment response, tolerability, and patient preferences. This is particularly important in IGM, where both overtreatment and undertreatment may have important consequences.

Future research should address the major weaknesses of the current evidence base. Prospective comparative studies are needed to evaluate the relative benefits and harms of systemic corticosteroids, local steroid therapy, surgery, steroid-based combinations, and methotrexate-based regimens. Standardized definitions of remission, recurrence, treatment failure, and recurrent or refractory disease should be adopted to improve comparability across studies. Future studies should also incorporate standardized clinical classification or severity stratification systems, particularly when evaluating surgical treatment or recurrent and refractory IGM. In addition, cosmetic outcomes, postoperative breast appearance, patient satisfaction, quality of life, and treatment burden should be systematically reported, especially in studies evaluating surgical or multimodal treatment strategies. Finally, the distinction between strictly defined IGM and broader granulomatous mastitis populations should be preserved in future evidence syntheses, as mixing these populations may reduce interpretability and clinical applicability.

## 5 Conclusion

This umbrella review suggests that steroid-based combination therapy shows the most consistent favorable signal, particularly for recurrence reduction. In contrast, local steroid therapy and methotrexate-based therapy appear promising in selected clinical contexts. Nevertheless, the overall certainty of the evidence remains limited because of methodological weaknesses in the included reviews and the observational nature of much of the underlying literature. Further high-quality comparative research is needed before firm treatment recommendations can be established.

## Data Availability

The original contributions presented in this study are included in the article/Supplementary material, further inquiries can be directed to the corresponding author.

## Supplementary material

Supplementary Table 1 Detailed search strategies for all databases.

Supplementary Table 2 AMSTAR-2 appraisal of the included reviews.

Supplementary Table 3 Citation matrix of primary studies across the included reviews.
