# Supplementary material for: Therapeutic strategies for idiopathic granulomatous mastitis: an umbrella review of systematic reviews and meta-analyses
Source: Front Med (Lausanne). 2026 Jun 3;13:1852206. doi: 10.3389/fmed.2026.1852206 (PMC13272329; doi:10.3389/fmed.2026.1852206)
Supplement: Supplementary Table 3 — Citation matrix of primary studies across the included reviews. [file Table_3.docx]

**1、PUBMED**

(

"Idiopathic Granulomatous Mastitis"[Title/Abstract]

OR "Granulomatous Mastitis"[Title/Abstract]

OR "Granulomatous Lobular Mastitis"[Title/Abstract]

OR "Idiopathic Granulomatous Lobular Mastitis"[Title/Abstract]

OR "Mastitis, Granulomatous"[MeSH Terms]

)

AND

(

treatment[Title/Abstract]

OR therapy[Title/Abstract]

OR therapies[Title/Abstract]

OR management[Title/Abstract]

OR surgery[Title/Abstract]

OR surgical[Title/Abstract]

OR corticosteroid*[Title/Abstract]

OR steroid*[Title/Abstract]

OR glucocorticoid*[Title/Abstract]

OR prednisolone[Title/Abstract]

OR prednisone[Title/Abstract]

OR methotrexate[Title/Abstract]

OR MTX[Title/Abstract]

OR antibiotic*[Title/Abstract]

OR drainage[Title/Abstract]

OR observation[Title/Abstract]

OR conservative[Title/Abstract]

OR intralesional[Title/Abstract]

OR local steroid[Title/Abstract]

)

AND

(

"systematic review"[Publication Type]

OR "meta-analysis"[Publication Type]

OR "systematic review"[Title/Abstract]

OR "meta-analysis"[Title/Abstract]

OR "meta analysis"[Title/Abstract]

OR "network meta-analysis"[Title/Abstract]

OR "network meta analysis"[Title/Abstract]

OR "review"[Title/Abstract]

)

1. **EMBASE**

(

'idiopathic granulomatous mastitis':ti,ab,kw

OR 'granulomatous mastitis':ti,ab,kw

OR 'granulomatous lobular mastitis':ti,ab,kw

OR 'idiopathic granulomatous lobular mastitis':ti,ab,kw

OR 'granulomatous mastitis'/exp

)

AND

(

treatment:ti,ab,kw

OR therapy:ti,ab,kw

OR management:ti,ab,kw

OR surgery:ti,ab,kw

OR surgical:ti,ab,kw

OR corticosteroid*:ti,ab,kw

OR steroid*:ti,ab,kw

OR glucocorticoid*:ti,ab,kw

OR prednisolone:ti,ab,kw

OR prednisone:ti,ab,kw

OR methotrexate:ti,ab,kw

OR MTX:ti,ab,kw

OR antibiotic*:ti,ab,kw

OR drainage:ti,ab,kw

OR observation:ti,ab,kw

OR conservative:ti,ab,kw

OR intralesional:ti,ab,kw

OR 'local steroid':ti,ab,kw

)

AND

(

'systematic review':ti,ab,kw

OR 'meta analysis':ti,ab,kw

OR 'meta-analysis':ti,ab,kw

OR 'network meta-analysis':ti,ab,kw

OR 'network meta analysis':ti,ab,kw

OR 'systematic review'/de

OR 'meta analysis'/de

)

AND [english]/lim

1. **Web of Science Core Collection**

**TS=(**

**("idiopathic granulomatous mastitis"**

**OR "granulomatous mastitis"**

**OR "granulomatous lobular mastitis"**

**OR "idiopathic granulomatous lobular mastitis")**

**AND**

**(treatment**

**OR therapy**

**OR management**

**OR surgery**

**OR surgical**

**OR corticosteroid***

**OR steroid***

**OR glucocorticoid***

**OR prednisolone**

**OR prednisone**

**OR methotrexate**

**OR MTX**

**OR antibiotic***

**OR drainage**

**OR observation**

**OR conservative**

**OR intralesional**

**OR "local steroid")**

**AND**

**("systematic review"**

**OR "meta-analysis"**

**OR "meta analysis"**

**OR "network meta-analysis"**

**OR "network meta analysis")**

**)**

1. **Cochrane Library**

**(**

**"idiopathic granulomatous mastitis"**

**OR "granulomatous mastitis"**

**OR "granulomatous lobular mastitis"**

**OR "idiopathic granulomatous lobular mastitis"**

**):ti,ab,kw**

**AND**

**(**

**treatment**

**OR therapy**

**OR management**

**OR surgery**

**OR surgical**

**OR corticosteroid***

**OR steroid***

**OR glucocorticoid***

**OR prednisolone**

**OR prednisone**

**OR methotrexate**

**OR MTX**

**OR antibiotic***

**OR drainage**

**OR observation**

**OR conservative**

**OR intralesional**

**OR "local steroid"**

**):ti,ab,kw**

**AND**

**(**

**"systematic review"**

**OR "meta-analysis"**

**OR "meta analysis"**

**OR "network meta-analysis"**

**OR "network meta analysis"**

**OR review**

**):ti,ab,kw**
